# Fire records in glacier ice

**DOI:** 10.1093/nsr/nwy159

**Published:** 2018-12-20

**Authors:** Chao You, Tandong Yao

**Affiliations:** 1 Key Laboratory of Tibetan Environment Changes and Land Surface Processes, Chinese Academy of Sciences, China; 2 CAS Center for Excellence in Tibetan Plateau Earth Sciences, China

## INTRODUCTION

Fire, sometimes called biomass burning, is a key factor affecting many aspects of the Earth system, including climate change, ecosystems, land surface processes, the carbon cycle, atmospheric chemistry and human society [1–3]. Understanding fire dynamics can provide insights into the interactions between fire and these components. Satellite data can provide high spatial-temporal resolution of fire changes for the most recent decades, while some historical information—such as the area burned in a certain region—can be traced back to the beginning of the twentieth century [1]. Fire changes over longer time scales (historical to ancient) can be derived from proxy records in the lithosphere, biosphere, cryosphere, hydrosphere and anthroposphere [1,3]. A combination of several evidence sources can yield a good estimate of the true fire variability [1–4].

Charcoal fossils in sediments can provide fire information since the Silurian (about 420 million years ago) [1], potentially yielding detailed evidence of fire distribution, intensity and frequency [1,3]. The resolution of such records is decadal at best, and some have been disturbed by human activities over the past few decades to several centuries in densely populated regions such as subtropical Asia [3]. Fire changes, and their influences on the environment and human society, are an important issue because the recent warming and drying trend has been reported to favor increased fire frequency [5]. High-resolution reconstructions of ancient fires in glacier ice are therefore crucial, and will constrain modeling predictions and inform policy making [1,3].

## FIRE TRACERS IN GLACIER ICE

The normal focus of fire studies in glacier ice is the chemical components, including characteristic ions (e.g. ammonium), black carbon, trapped air (e.g. methane and carbon monoxide), low-molecular-weight organic acids (e.g. formate and oxalate), persistent organic pollutants, resinic acids and monosaccharide anhydrides (e.g. levoglucosan) [2]. Each of these components provides a different line of evidence [2,4]. The first study of fire records in glacier ice, by Legrand and colleagues, used the Greenland GRIP ice core [2,6]. Scientists found that large increases in ammonium concentrations in some specific ice layers were associated with elevated levels of formate, acetate, oxalate and some other proxies, and claimed that these chemical signatures were due to fire events across the Arctic and its surrounding regions [6]. Since then, electrical conductivity measurements, black carbon, vanillic acids and trapped air bubbles have provided powerful evidence for studying the changing characteristics of past fire emissions [2].

These chemical species often have multiple potential sources in addition to fire emissions [2,4] and consequently more specific tracers—related only to fire emissions—are preferred. Levoglucosan can only be generated from the degradation of cellulose and hemicellulose when the combustion temperature is higher than 300°C [4]. The lifetime of levoglucosan varies from several hours to more than 10 days under different atmospheric conditions, and thus ensures its long-range transport and global distribution [4,7–9]. Levoglucosan has been widely detected in the Antarctic and Greenland ice sheets, and in mid- to low-latitude mountain glaciers [2,4,5,7–9]. Furthermore, levoglucosan displays almost no apparent degradation in the freezing and anaerobic conditions in glacier ice layers [8], thus possibly enabling its use as a specific biomarker for ancient fires in ice records [5,8–10].

## HIGH-RESOLUTION FIRE RECORDS

Due to the long-distance transport, fire signals detected in glacier ice are usually at extremely low levels when compared to those of the source regions [4]. Although post-depositional photochemical or leaching processes can modify the records of organic compounds on glacier surfaces, it should be noted that they can be used as proxies for fire changes at least on seasonal to annual scales over the accumulation zone, in either polar ice sheets or mountain glaciers [4,8]. Furthermore, some tracers can even capture event-based signals [4,8]. For instance, the ad 1994 summer forest fire events over the Canadian Arctic regions were detected by ammonium, formate, levoglucosan and oxalate records in Greenland snow layers [8]. Similarly, years characterized by extreme fire events (ad 1908, 1895, 1863, etc.) were identified by abnormal peaks of ammonium, formate, glycolate, oxalate and acetate in Greenland ice cores [2]. Nevertheless, there is not a one-to-one relationship between fire events in the source region and fire records detected in glacier ice [4].

Polar ice sheets can provide more detailed information on fire after the Last Glacial Period [2,6], and in particular can provide seasonal to annual reconstructions covering the past millennium [9] to the past few decades [5]. High concentrations of carboxylic acids in a Greenland ice core indicated that more frequent biomass burning events occurred during the warm stage than during the cold stage over the last glacial cycle period [2,9]. Sudden warming events resulted in an increase in the frequency of wildfires over North America, as deduced from ammonium records in Greenland NGRIP and GRIP ice cores [2]. Similarly, higher levoglucosan concentrations were reported in inter-glacial ice than in glacial ice in the Dome C ice core from Antarctica [4]. Levoglucosan records reconstructed from both Greenland and Antarctic ice cores have revealed significant increases since the last glacial, reaching a maximum around 2500 years before the present, then decreasing until the present; these changes may have been caused by anthropogenic activities [9,10]. Some periods of high fire activities were detected by peaks in levoglucosan, vanillic acid and p-hydroxybenzoic acid concentrations over the past millennium. Fire changes over the past few decades remain as a controversial issue across high-latitude regions [2], and further high-resolution evidence from glacier ice is needed to resolve this dispute [2,3].

Alpine glacier ice can provide some essential information about fire changes on regional scales [5,7]. The fire regime over southern Siberia since ad 1250 was reconstructed using nitrate, potassium and charcoal records in the Belukha ice core from the Siberian Altai; these records suggested that precipitation changes were the driving factor controlling fire changes. Some sporadic fire events have been reported in glacier ice on the Tibetan Plateau [5] and Kamchatka peninsula [7]. Fires increased at around 1980, followed by a decrease in the 1990s, as deduced from levoglucosan and black carbon records in ice cores from the Muztag Ata and the Caucasus—both of which are affected by the middle-latitude westerlies. Intensified fire emissions due to anthropogenic activities were detected during the 1940s to 1950s [2,4]. In comparison, levoglucosan records revealed that weakened rainfall due to changes in the Indian summer monsoon in the early twenty-first century resulted in a rapid increase in wildfires across the Himalayas and surrounding regions—an event that was independent of longer-term trends [5].

## PERSPECTIVE FOR FUTURE WORKS

To better understand ancient fire changes reconstructed in glacier ice, several associated issues need further attention in future work.

The most notable issue regards the geochemical significance of fire tracers. A large concentration of photochemically active groups is present in the snowpack [2,4], which could potentially degrade biomarkers after deposition [4]. Much stronger evidence is needed to validate the stability of these tracers in glacier ice. Post-depositional processes, such as leaching and wind scour, on glacier surfaces can also reshape the initial records [2,8]. Such processes might affect the reliability of any reconstructed fire records, yet their impacts remain poorly understood [2,4]. Therefore, combining biomarkers with traditional proxies (e.g. black carbon) is necessary to achieve more confidence in fire reconstructions.

Challenges in tracing fire sources are also an urgent issue. Those based on back trajectories can only provide rough results [5,7–10] and more precise results should be obtained using the isotopic or isomeric methods. Burning of various biomass types can result in different proportions of levoglucosan, mannosan and galactosan [4]. With the continued development of analytical methods, we can potentially detect these three isomers at trace (ng g^−1^) or ultra-trace (pg g^−1^) concentrations or even lower [4], which is helpful for identifying fire source regions. Furthermore, developing greater sensitivity in new proxies is important, because a large number of unknown chemical components (mostly organics) exist in glacier ice [2,4].

Precise dating of ice cores is critical for capturing the detailed characteristics of fire variations. Obtaining continuous results can reveal the frequency of fire events as well as longer-term temporal changes [5], especially from the multi-decadal to millennium scales. Besides, continuous results can potentially improve our understanding of the causes of differences between records obtained from various proxies [2]. In addition, some specific years with extreme fire events could be used as absolute layers for calibrating ice-core dating results in the future, notably for those ice cores from alpine regions that usually lack absolute layers for cross-validation. Indeed, glacier ice in low- and middle-latitude alpine regions can yield valuable data for better understanding fire variations, particularly in densely populated regions (e.g. subtropical Asia). The recent, rapid retreat of high-elevation alpine glaciers makes collecting such records an urgent objective in low- and mid-latitude regions.
